# Saikosaponin A inhibits influenza A virus replication and lung immunopathology

**DOI:** 10.18632/oncotarget.6448

**Published:** 2015-12-02

**Authors:** Jianxin Chen, Mubing Duan, Yaqin Zhao, Fangfang Ling, Kun Xiao, Qian Li, Bin Li, Chunni Lu, Wenbao Qi, Zhenling Zeng, Ming Liao, Yahong Liu, Weisan Chen

**Affiliations:** ^1^ Guangdong Provincial Key Laboratory of Veterinary Pharmaceutics Development and Safety Evaluation, College of Veterinary Medicine, South China Agricultural University, Guangzhou, China; ^2^ Department of Biochemistry and Genetics, La Trobe Institute for Molecular Science, La Trobe University, Melbourne, Victoria, Australia; ^3^ National Engineering Research Center of Immunological Products, Department of Microbiology and Biochemical Pharmacy, College of Pharmacy, Third Military Medical University, Chongqing, China; ^4^ Present address: Xinjiang Institute of Chinese Materia Medica and Ethnic Materia Medica, Urumqi, Xinjiang, China

**Keywords:** Saikosaponin A, anti-inflammatory agent, influenza A virus, PR8, X-31, Immunology and Microbiology Section, Immune response, Immunity

## Abstract

Fatal influenza outcomes result from a combination of rapid virus replication and collateral lung tissue damage caused by exaggerated pro-inflammatory host immune cell responses. There are few therapeutic agents that target both biological processes for the attenuation of influenza-induced lung pathology. We show that Saikosaponin A, a bioactive triterpene saponin with previouslyestablished anti-inflammatory effects, demonstrates both *in vitro* and *in vivo* anti-viral activity against influenza A virus infections. Saikosaponin A attenuated the replication of three different influenza A virus strains, including a highly pathogenic H5N1 strain, in human alveolar epithelial A549 cells. This anti-viral activity occurred through both downregulation of NF-κB signaling and caspase 3-dependent virus ribonucleoprotein nuclear export as demonstrated by NF-κB subunit p65 and influenza virus nucleoprotein nuclear translocation studies in influenza virus infected A549 cells. Critically, Saikosaponin A also attenuated viral replication, aberrant pro-inflammatory cytokine production and lung histopathology in the widely established H1N1 PR8 model of influenza A virus lethality in C57BL/6 mice. Flow cytometry studies of mouse bronchoalveolar lavage cells revealed that SSa exerted immunomodulatory effects through a selective attenuation of lung neutrophil and monocyte recruitment during the early peak of the innate immune response to PR8 infection. Altogether, our results indicate that Saikosaponin A possesses novel therapeutic potential for the treatment of pathological influenza virus infections.

## INTRODUCTION

Influenza A virus (IAV) infection remains a global health burden due to both seasonal IAV disease susceptibilities in young, chronically afflicted and elderly populations and the potential re-emergence of highly fatal pandemic IAV strains which do not respond to current treatments. Fatality rates from seasonal IAV strains are the highest within elderly (> 65 years) and young (< 5 years) age groups [1, 2] whereas otherwise healthy young adults are disproportionately affected by fatal pandemic IAV infections [3]. Although vaccination strategies are well-established for influenza prevention and control, seasonal IAV vaccines exhibit decreased efficacies in elderly individuals [4] and a prophylactic response against pandemic IAV strains can be delayed by lengthy vaccine production cycles. Antiviral agents are the current standard for primary care in highly susceptible individuals, although treatment efficacy decreases with delayed administration times [5] and sporadic resistance is possible [6, 7]. Thus, the identification of novel anti-IAV agents against influenza induced disease pathology remains an urgent healthcare priority.

Severe pneumonia is a common cause of primary IAV-related morbidities and mortalities, especially for pandemic IAV strains. IAV infection begins in the upper respiratory track epithelial cells [8] and spreads into the deeper regions of the lung parenchyma [9]. At the same time, pro-inflammatory responses are initiated by the host and involve the production of pro-inflammatory cytokines (IFN-α, IFN-γ, TNF-α and IL-6) and chemokines (KC, MIP-1α, MIP-2, CCL2, CCL5, CXCL10) which facilitate the initial phase of innate immune cell recruitment into the lungs, typically from day 1 to 5 post-infection [10]. Although pro-inflammatory responses are critical for the early control of viral replication [11], excessive inflammation also increases the level of tissue damaging cytotoxic and pro-apoptotic products in the lungs [12]. In particular, heightened lung neutrophil and monocyte numbers have been causally linked to the development of severe pneumonia in mouse models of lethal IAV infections [13, 14], although complete abrogation of these immune cells also profoundly impairs host IAV clearance [11, 14]. Thus, novel anti-IAV pharmaceutical agents must navigate a critical balance between the engagement of sufficient innate immune cell signaling to control IAV propagation and the attenuation of excessive pro-inflammatory and tissue damaging effectors.

NF-κB signaling is rapidly initiated in response to pathogen-associated molecular patterns (PAMPs) sensing and promotes the transcription of a diverse array of genes including pro-inflammatory cytokines and chemokines, immunomodulatory growth factors and cell surface receptors [15]. Due to its rapid ability to induce gene transcription, NF-κB signaling is often simultaneously appropriated by viral pathogens including IAV to enhance viral replication efficiencies [16]. Interestingly, host cell NF-κB activation is a prerequisite for IAV virus replication as both pharmacological inhibition and siRNA-mediated gene silencing of NF-κB signaling decreases IAV propagation *in vitro* [17–19]. Given that NF-κB signaling is also responsible for the aberrant pro-inflammatory responses associated with fatal cases of IAV infection [20], downregulation but not complete inhibition of this signaling pathway may provide a novel ‘dual-hit’ strategy for the attenuation of IAV-related lung pathology.

**Figure 1:**
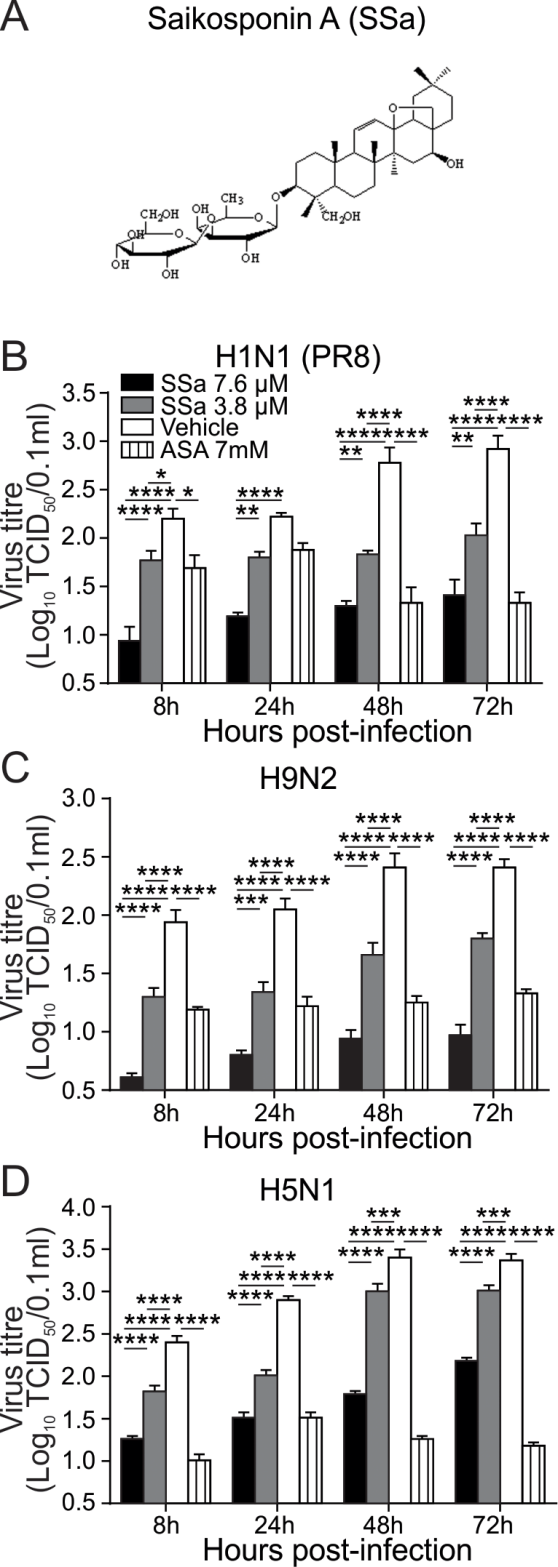
SSa attenuates IAV replication in A549 cells **A.** Chemical structure of SSa. A549 cells were infected with **B.** A/Puerto Rico/8/34 (H1N1 PR8), **C.** A/Chicken/Guangdong/v/2008 (H9N2), or **D.** A/Duck/Guangdong/99 (H5N1) IAV strains at 100 TCID_50_ for 2 h and then incubated with 3.8 or 7.6 μM SSa or 7 mM acetylsalicylic acid respectively. Cells and supernatants were harvested at 8, 24, 48 and 72 h post-infection and total IAV yield calculated by TCID_50_ titration using MDCK cells. Data represented as mean ± SEM of three independent experiments. *P < 0.05, ^**^P < 0.01, ^***^P < 0.001, ^****^P < 0.0001.

Saikosaponin A (SSa) is one of the major bioactive triterpene saponins derived from the root of *Bupleurum chinense DC* and constitutes approximately 100-300 mg/g of its total chemical composition [21]. Other bioactive constituents of *Radix Bupleurum* include Saikosaponins B, C and D (closely related triterpenoidal structures with differing carbohydrate attachments to SSa), the pectic polysaccharide Bupleuran 2IIc [22] and several lignans, flavonoids and essential oils [23]. Crude extracts of *Radix Bupleurum* have been historically prescribed as a supportive treatment for acute respiratory infection-associated pyrexia and analgesia as well as for chronic hepatitis and some autoimmune diseases [23]. Given its steroid-like chemical structure (Figure 1A) and reported NF-κB inhibiting anti-inflammatory activities *in vitro* and *in vivo* [24–27], we conducted a comprehensive analysis of the effects of SSa on IAV propagation and lung immunopathogenesis, especially in relation to NF-κB signaling associated cellular pathways. SSa attenuated the replication of three different IAV strains, including a highly pathogenic H5N1 strain, in A549 cells through downregulation of NF-κB signaling and caspase 3-dependent virus ribonucleoprotein nuclear export. Critically, Saikosaponin A also attenuated viral replication, aberrant pro-inflammatory cytokine production and lung neutrophil and monocyte recruitment in the PR8 model of influenza lethality in C57BL/6 mice.

## RESULTS

### SSa inhibits IAV replication in A549 cells

SSa cytotoxicity was first established using the MTT viability assay on A549 cells and through monitoring of body weight changes and adverse symptoms in SSa treated B6 mice (Supplementary Figure 1). Minimal cytotoxicity was observed for SSa concentrations ≤ 7.6 μΜ 48 h post-treatment on A549 cells *in vitro* (Supplementary Figure 1A) and 7.6 μΜ SSa hence selected as the maximal drug concentration used for subsequent IAV infection studies. Saikosaponin D, an epimer of SSa that also downregulates LPS-induced NF-κB signaling [26], showed heightened cytotoxicity levels in A549 cells compared to SSa (data not shown) and was not therefore further investigated in our study. To investigate for potential *in vitro* anti-IAV activity, we first evaluated the effects of SSa on IAV propagation in A549 cells. A549 cells were infected with three different IAV strains of different pathogenicity levels (H1N1 PR8, H9N2 and high pathogenicity H5N1) for 8, 24, 48 and 72 h post-infection and total cell and supernatant virus titres calculated. Acetylsalicylic acid (ASA; aspirin) has been reported to inhibit *in vitro* and *in vivo* IAV viral replication through downregulation of NF-κB signaling [18] and was used as a positive control. IC_50_ values for the inhibition of IAV replication were 1.98, 2.21 and 2.07 μM for H1N1 PR8, H9N2 and H5N1 strains respectively. For all three IAV strains, SSa inhibited IAV replication in a dose-dependent manner from 8, 24, 48 and 72 h post-infection similar to the inhibitory effects of ASA (Figure 1B–1D).

### SSa suppresses IAV-induced NF-κB activation in A549 cells

**Figure 2:**
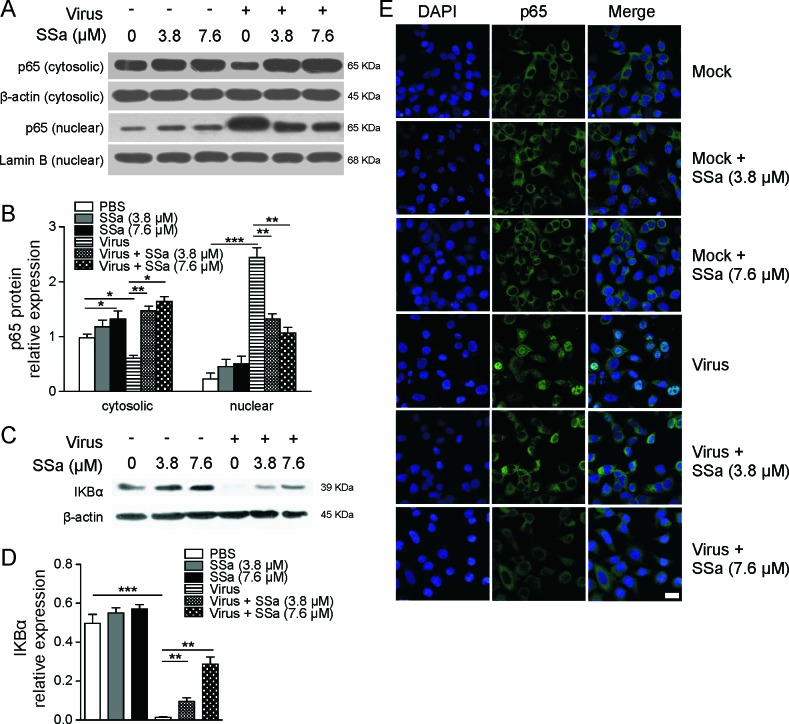
SSa inhibits nuclear NF-κB protein translocation in H5N1 infected A549 cells **A.** and **B.** A549 cells infected with H5N1 IAV (MOI = 0.1, 1 h) and cultured ± 3.8 or 7.6 μM SSa were harvested 24 h post-infection. Non-infected A549 cells were also cultured ± 3.8 or 7.6 μM SSa and harvested at the same time point. **A.** Cytosolic and nuclear immunoblotting was conducted for NF-κB p65 subunit expression with lamin B1 (nuclear) and β-actin (cytosolic) loading controls. Results are representative of three independent immunoblotting experiments. **B.** Relative gray scale values of cytosolic and nuclear p65 expression from three independent immunoblotting assays as depicted in **A.**. *P < 0.05, ^**^P < 0.01. **C.** Immunoblotting of A549 cells lysed at 24 h post-infection for total protein IκBα expression with β-actin as a loading control. Non-infected A549 cells were also cultured ± 3.8 or 7.6 μM SSa and harvested at the same time point. Results are representative of three independent immunoblotting experiments. **D.** Relative gray scale values of total protein IκBα expression quantified from **C.** *P < 0.05. **E.** H5N1-infected A549 cells (MOI = 1, 1 h) were cultured ± 3.8 or 7.6 μM SSa for 8 h post-infection, fixed and stained for p65 expression (p65: green, DAPI nuclear stain: blue, scale = 10 μm). Representative of three independent experiments. Non-infected ± 3.8 or 7.6 μM SSa treated A549 cells were visualised in parallel.

During IAV infection, NF-κB is appropriated by IAV for productive host cell infection and active NF-κB signaling is required for IAV propagation itself [17]. To investigate whether SSa-mediated inhibition of IAV replication was dependent on NF-κB signaling in high pathogenicity H5N1 IAV infections, we examined the effects of SSa treatment on NF-κB nuclear translocation in H5N1-infected A549 cells. At steady state, NF-κB predominantly exists as a heterodimer of RelA (p50) and p65 subunits bound by an IκB inhibitor protein (usually IκBα or IκBβ) which prevents nuclear translocation and gene transcription [28]. Activation of NF-κB signaling is mediated by the phosphorylation and degradation of IκB by the IκB kinase complex, facilitating nuclear translocation of RelA/p65 subunits where they bind to NF-κB promoter regions and initiate gene transcription [28]. As expected, H5N1 infection greatly increased nuclear but not cytosol p65 levels at 24 h post-infection compared to non-infected controls, demonstrating IAV-induced activation of NF-κB signaling (Figure 2A and 2B). SSa treatment attenuated IAV-induced nuclear p65 translocation (Figure 2A and 2B). Consistent with attenuated p65 nuclear translocation, SSa treatment also decreased the level of IAV-induced IκBα degradation in infected A549 cells (Figure 2D and 2E). Immunofluorescence studies further confirmed decreased p65 nuclear translocation in SSa treated H5N1-infected A549 cells at 8 h post-infection compared to untreated controls (Figure 2D). SSa addition itself did not affect p65 nuclear translocation similar to non-infected (mock) A549 cells (Figure 2D) although a modest but statistically significant increase in cytosolic p65 expression was observed at 7.6 μM.

### SSa attenuates nuclear export of viral ribonucleoproteins in A549 cells

**Figure 3:**
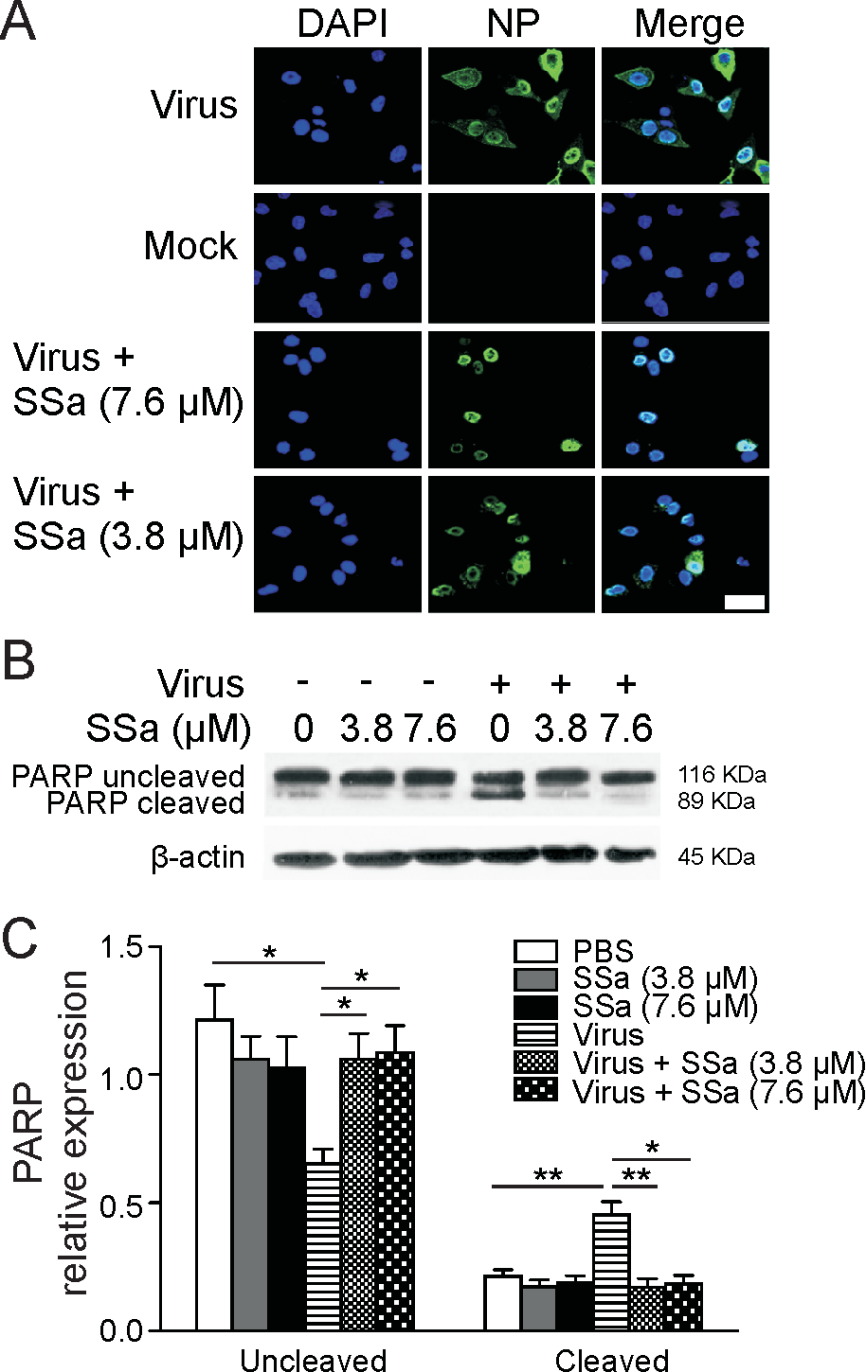
SSa attenuates vRNP export in H5N1-infected A549 cells **A.** H5N1-infected A549 cells (MOI = 1, 1 h) were cultured ± 3.8 or 7.6 μM SSa for 8 h post-infection, fixed and stained for NP expression (NP: green, DAPI nuclear stain: blue, scale = 50 μm). Representative of three independent experiments. **B.** Immunoblotting of H5N1-infected A549 cells (MOI = 0.1, 1 h) cultured ± 3.8 or 7.6 μM SSa and lysed 24 h post-infection using an anti-PARP polyclonal antibody with β-actin as a loading control. Representative of three independent experiments. Non-infected A549 cells were also cultured ± 3.8 or 7.6 μM SSa and harvested at the same time point. Representative of three independent experiments. **C.** Relative gray scale values of total uncleaved and cleaved PARP protein expression quantified from **B.**. *P < 0.05.

Nuclear export of newly assembled viral ribonucleoproteins (vRNPs) into the cytosol is required for the final assembly and release of IAV progeny virions [29, 30]. Interestingly, this process is dependent on pro-apoptotic caspase 3 activation [31, 32]. Cytosolic transport of vRNPs, reflected by cytosolic IAV nucleoprotein (NP) staining, was observed at 8 h post-H5N1 infection in A549 cells (Figure 3A). Cytosolic NP levels were decreased following SSa treatment 8 h post-IAV infection compared to untreated controls. (Figure 3A). Consistent with published findings, IAV infected MCF-7 cells that lack functional caspase 3 showed negligible cytosolic NP transport in the absence or presence of SSa (Supplementary Figure 2). Poly-(ADP-ribose)-polymerase (PARP) is a classical substrate of caspase 3 and PARP cleavage was increased following H5N1 infection, suggesting increased caspase activity (Figure 3B). SSa treatment decreased PARP cleavage in H5N1-infected A549 cells (Figure 3B). Cleaved (active) caspase 3, however, was not detectable by western blotting even in H5N1-infected positive controls (data not shown), which may reflect low protein yields or the immortalized properties of A549 cells.

### SSa attenuates IAV-induced mortality and lung pathogenesis following lethal PR8 infection in mice

SSa cytotoxicity was assessed *in vivo* and minimal weight loss and adverse symptoms (signs of hunching, unresponsiveness, piloerection, altered respiratory rates and alopecia) were observed in C57BL/6 (B6) mice administered 50 mg/kg/d compared to 100 and 200 mg/kg/d of SSa subcutaneously for 6 consecutive days (Supplementary Figure 1B). 50 mg/kg/d of SSa was therefore chosen as the maximal drug dose administered to IAV-infected B6 mice.

**Figure 4:**
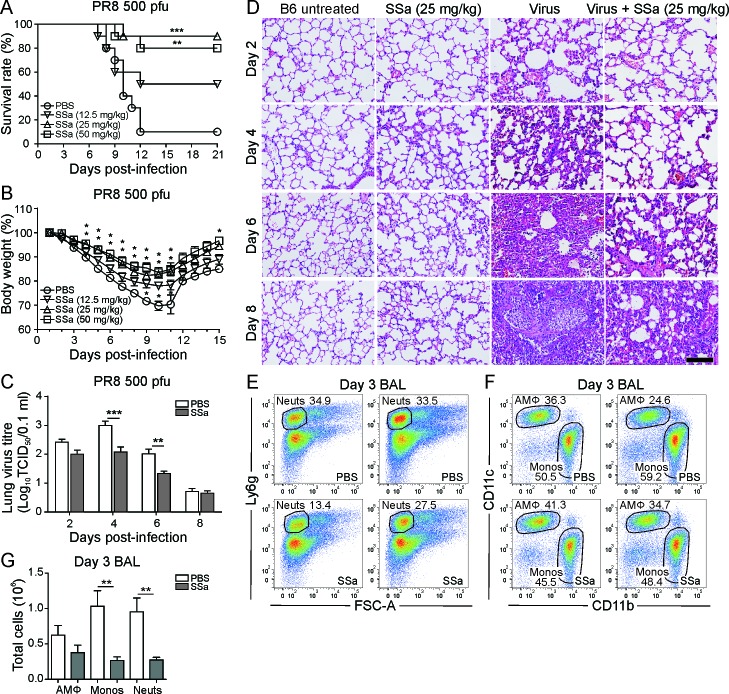
SSa protects against PR8 induced morbidity and mortality in B6 mice **A.** B6 mice infected with 500 pfu of PR8 and treated with SSa or PBS control (daily subcutaneous injections for a consecutive 6 days beginning at 4 h post-infection). Effect of SSa administration on survival rates in PR8-infected B6 mice (*n* = 10) **P < 0.01 and ***P < 0.001 with respect to PBS controls. **B.** Effect of SSa administration on body weight loss in PR8-infected B6 mice (*n* = 10). ^*^ denotes *P* < 0.05 or lower for 50 mg/kg/d (top), 25 mg/kg/d (middle) and 12.5 mg/kg/d (bottom) with respect to PBS controls. **C.** Effect of SSa administration (25 mg/kg/d) on lung virus titres in PR8-infected B6 mice (*n* = 5). **P < 0.01 and ***P < 0.001 with respect to PBS controls. **D.** H&E lung sections of PR8-infected and non-infected B6 mice treated with SSa or PBS controls (representative images from *n* = 3 per group, scale = 100 μm). All data represented as mean ± SEM. **E.** Total proportion of Ly6g^high^FSC-A^low^ BAL neutrophils out of all CD45^pos^ singlet cells in SSa (25 mg/kg/d) *versus* PBS control treated B6 mice at day 3 post-PR8 infection (*n* = 7). Representative of two independent experiments. **F.** Total proportion of CD11c^high^CD11b^low/neg^ AMΦs and CD11c^low^CD11b^high^ lung monocytes out of all CD45^pos^ non-neutrophil and non-lymphocyte singlet cells in SSa (25 mg/kg/d) *versus* PBS control treated B6 mice at day 3 post-PR8 infection (*n* = 7). Representative of two independent experiments. **G.** Total BAL AMΦs, lung monocyte and neutrophil numbers in SSa (25 mg/kg/d) *versus* PBS control treated B6 mice at day 3 post-PR8 infection, with cell proportions calculated by flow cytometry (*n* = 7). **P < 0.01 with respect to PBS controls.

PR8 is a mouse-adapted IAV strain widely used to model lethal IAV infections and causes up to 90% mortality in B6 mice within 10 days following infection at 500 pfu/mouse. B6 mice treated with 25 mg/kg/d and 50 mg/kg/d SSa showed significantly decreased mortality rates compared to 12.5 mg/kg/d SSa and PBS control groups following infection with 500 pfu of PR8 (Figure 4A). This correlated with a corresponding reduction in weight loss following PR8 infection in 25 mg/kg/d and 50 mg/kg/d SSa treated B6 mice (Figure 4B) and 25 mg/kg/d was chosen as the optimal treatment dose for all further IAV studies as 50 mg/kg/d SSa still elicited modest weight loss in B6 mouse toxicity studies (Supplementary Figure 1B). Similar to *in vitro* findings, SSa administration decreased total lung tissue viral titres at day 4 and 6 post-PR8 infection (Figure 4C). Critically, lung histology showed an attenuation of inflammatory infiltrates in the parenchyma, airways and interstitial regions of the lungs following SSa treatment in PR8-infected B6 mice (Figure 4D). SSa treatment (25 mg/kg/d) alone did not induce any pathological changes in lung tissue compared to that of untreated B6 mice or treated PBS controls (Figure 4D). As ASA inhibited IAV virus replication *in vitro* similarly or more effectively than SSa, we also examined the effects of ASA administration following lethal PR8 infections in B6 mice (Supplementary Figure 3A). Interestingly, ASA did not significantly reduce PR8-induced morbidity or mortality compared to SSa, suggesting that SSa efficacy may also be potentially attributed to other anti-inflammatory effects rather than anti-viral activity alone (Supplementary Figure 3B and 3C). SSa administration 24 h prior to and 24 h but not 72 h following PR8 infection was also protective against PR8-induced mortality in B6 mice (Supplementary Figure 3D), suggesting early control of IAV replication and/or tissue damaging mediators as potential mechanisms of SSa-mediated anti-IAV protection.

### SSa selectively attenuates lung neutrophil and monocyte recruitment following lethal PR8 infection in mice

We therefore investigated the potential effects of SSa treatment on early phase lung immune cell recruitment in PR8-infected B6 mice. Lung neutrophils are the first immune cells recruited into the alveolar airspaces in response to IAV infection and peak in numbers around day 3 post-infection [33], when lung monocyte and natural killer (NK) cell recruitment also increases. SSa treatment significantly decreased bronchoalveolar lavage (BAL) neutrophil and monocyte but not AMФ numbers by day 3 post-infection compared to PBS controls (Figure 4E–4G). Differences in NK, CD4^+^ and CD8^+^ T cell numbers were not statistically significant, although a trend towards decreased NK cell recruitment was also observed in SSa treated PR8-infected mice (Supplementary Figure 4A). The attenuation of lung neutrophil and monocyte recruitment in SSa treated mice was not due to a systemic suppression of neutrophil or monocyte production (Supplementary Figure 4B–4G) with bone marrow neutrophil numbers conversely increased in SSa treated mice at day 3 post-PR8 infection. No differences existed in BAL neutrophil and monocyte CD11b or CD11a surface expression either, indicating that impairment of endothelial cell transmigration was not responsible for the reduction in SSa-mediated BAL neutrophil and monocyte numbers. To confirm that the immunomodulatory effects of SSa treatment were not confined to PR8 infection itself, we also investigated its effects on influenza A/X-31 (X-31) infected B6 mice at day 3 post-infection (Supplementary Figure 5). SSa treatment ameliorated X-31-induced weight loss in B6 mice (Supplementary Figure 5A) and this was associated with a selective reduction in BAL neutrophil numbers (Supplementary Figure 5B and 5C). A trend for decreased monocyte recruitment was also observed, although this did not reach statistical significance (Supplementary Figure 5B and 5C).

### SSa attenuates pro-inflammatory cytokine production following lethal PR8 infection

**Figure 5:**
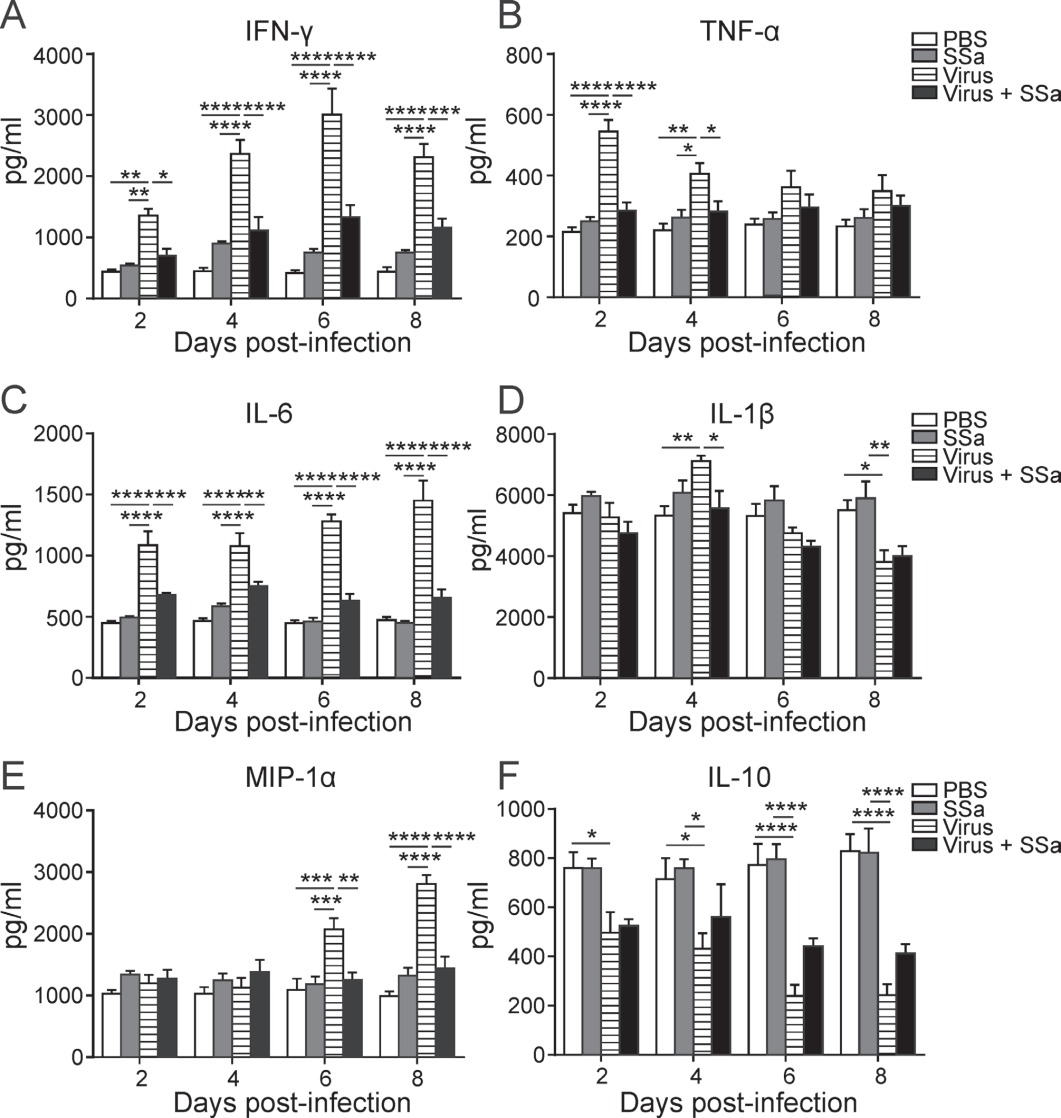
SSa treatment attenuates lung cytokine and chemokine production following PR8 infection in B6 mice B6 mice were infected with 500 pfu of PR8 and treated with SSa (daily subcutaneous injections of 25 mg/kg/d SSa for 6 consecutive days; *n* = 3 per group). BAL concentrations of **A.** IFN-γ, **B.** TNF-α, **C.** IL-6, **D.** IL-1β, **E.** MIP-1α and **F.** IL-10 were determined by ELISA. All data represented as mean ± SEM. *P < 0.05, **P < 0.01, ***P < 0.001and ****P < 0.001.

Heightened production of pro-inflammatory cytokines and chemokines is a hallmark of lethal IAV infections and correlates clinically with increased IAV pathogenicity [34–36]. SSa treatment reduced BAL IFN-γ production at all time points tested following PR8 infection (Figure 5A). During IAV infection, lung monocytes and monocyte-derived dendritic cells are also a steady source of pro-inflammatory cytokines such as TNF- α and IL-6 [14, 37]. In parallel with decreased lung monocyte infiltration, SSa treatment attenuated PR8-induced IL-6 production in the lungs at all time points studied and attenuated TNF-α production from days 2 and 4 following PR8 infection (Figure 5B–5C). A modest attenuation of IL-1β and MIP-1α also occurred at day 4 and from day 6 onwards respectively in SSa treated PR8-infected B6 mice (Figure 5D and 5E). No significant differences in IL-10 production were observed between SSa treated and PBS control mice at all time points following PR8 infection (Figure 5F). Parallel with lung histology findings, SSa administration alone did not alter the levels of IFN-γ, TNF-α, IL-6, IL-1β, MIP-1α and IL-10 present in the lungs of non-infected B6 mice (Figure 5).

### SSa attenuates lung immune cell apoptosis following lethal PR8 infection in mice

**Figure 6:**
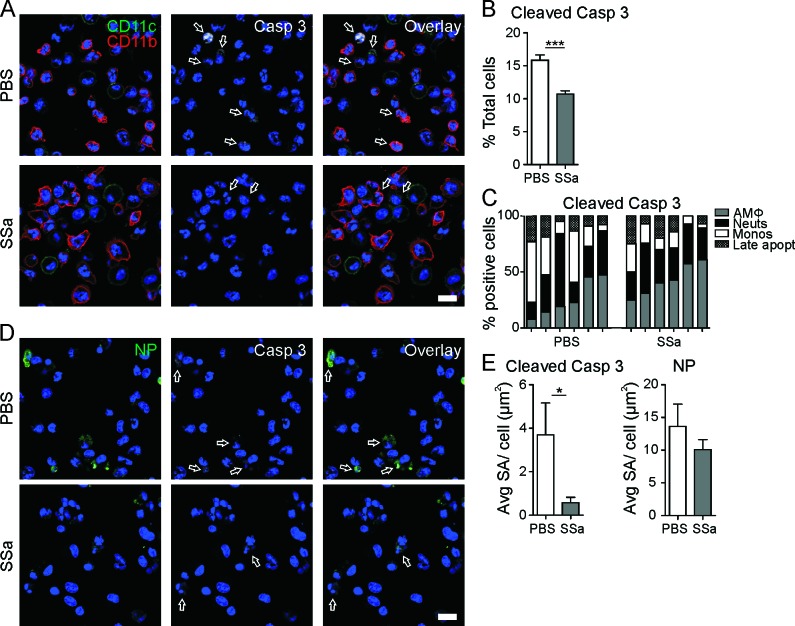
SSa decreases BAL immune cell caspase 3 and NP expression levels in PR8-infected B6 mice **A.** Immunofluorescence of cleaved caspase 3 expression (white) in BAL cells co-stained with surface CD11b (green) and CD11c (red) from day 3 PR8-infected B6 mice treated with SSa (25 mg/kg/d) or PBS control (blue: Hoechst 33342-stained nucleus; scale = 25 μm; *n* = 6). Representative of two independent experiments. **B.** Proportion of all BAL cells positive for cleaved caspase 3 from **A.** as quantified using Imaris (*n* = 6, minimal of 6 × 50 cells studied per sample). Representative of two independent experiments. *** P < 0.001 for SSa with respect to PBS control. **C.** Proportion of cleaved caspase 3^pos^ BAL cells from **A.** identified as either CD11c^neg^CD11b^pos^ multi-lobular neutrophils, CD11c^neg/low^CD11b^pos^ monocytes, CD11c^pos^CD11b^neg/low^ AMФs or late apoptotic cells (with indistinguishable surface CD11b and CD11c staining) in day 3 PR8-infected B6 mice administered SSa or PBS control (*n* = 6). **D.** IAV NP (green) and cleaved caspase 3 (white; white arrows) staining in BAL cells from day 3 PR8-infected B6 mice administered SSa or PBS oil emulsions (25 mg/kg/d; blue: Hoechst 33342-stained nucleus; scale = 15 μm). Representative of *n* = 3. **E.** Average surface area of cleaved caspase 3 and NP staining per positively stained cell from **D.** calculated using thresholding in Imaris (from an average of ≥ 100 cells measured per sample) in day 3 PR8-infected B6 mice administered SSa or PBS control (25 mg/kg/d; *n* = 3). * *P* < 0.05 for SSa with respect to PBS control.

Immune cell modulation can directly alter the consequences of IAV pathogenesis independent of anti-viral targeting [14, 38]. To examine whether SSa attenuated *in vivo* IAV propagation through similar functional pathways as demonstrated *in vitro*, active caspase 3 and cytosolic NP expression were also quantified in BAL cells from SSa and PBS control treated B6 mice at day 3 post-PR8 infection (Figure 6). Total cleaved (active) caspase 3 expression was attenuated in BAL cells from SSa treated B6 mice compared to PBS controls (Figure 6A and 6B). Interestingly, a higher proportion of lung monocytes were positive for active caspase 3 expression in PBS control compared to SSa treated PR8-infected mice (Figure 6C), suggesting that monocyte apoptosis may be more prominent in lethal IAV pathogenesis. Concordant with our *in vitro* observations and *in vivo* IAV titre studies, decreased cytosolic NP expression was also observed in BAL cells of SSa compared to PBS control treated mice (Figures 6D). A trend for decreased total cellular NP expression was also observed although this was not statistically significant between SSa and PBS control treated mice (Figure 6E). Critically, BAL cells with high NP expression were also frequently positive for active caspase 3 expression in PBS control but not SSa treated mice (Figure 6D).

**Figure 7:**
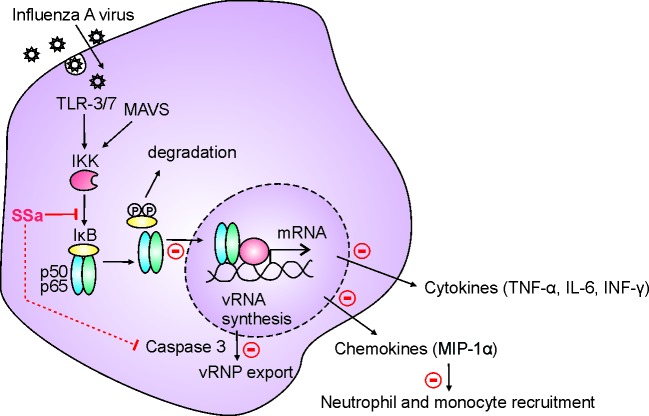
Schematic diagram of the mechanism of SSa-induced attenuation of IAV pathogenesis IAV infection triggers host cell activation of the NFκB signaling pathway following endosomal (TLR) and cytosolic (MAVS) IAV sensing. SSa directly inhibits IκB degradation and p65 nuclear translocation *in vitro*, decreases caspase 3-dependent IAV vRNP export *in vitro* and *in vivo* and attenuates NF-κB dependent pro-inflammatory cytokine production and lung neutrophil and monocyte recruitment *in vivo*.

Overall, we have demonstrated that SSa directly suppresses IAV propagation through inhibition of NF-κB signaling *in vitro* and is protective in the PR8 mouse model of IAV lethality. This occurred through the attenuation of NF-κB associated inflammatory pathways such as pro-inflammatory cytokine and chemokine release and downstream lung innate immune cell recruitment, as well as a modest decrease in caspase 3 associated IAV propagation (Figure 7).

## DISCUSSION

There are few therapeutic agents that can successfully ablate the progression of severe IAV-induced pneumonia. Patient mortality rates are approximately 60% even with anti-viral therapy and anti-viral drug resistant IAV mutations have been reported in patient subpopulations [6], prompting the need for alternative therapeutic strategies that do not directly target the virus and promote the selection of resistant IAV strains. Mechanistically, as heightened virus proliferation and pro-inflammatory innate immune responses are concurrently linked to the severity of IAV-induced lung pathogenesis [13, 35, 39], the discovery of new therapeutic agents that can indirectly inhibit IAV replication whilst attenuating tissue-damaging pro-inflammatory host responses remains a critical priority. The need to therapeutically fine-tune rather than completely abrogate this latter event is emphasised by recent animal studies showing unaltered or conversely increased IAV-induced mortality rates following complete removal of pro-inflammatory cytokines, lung neutrophils or monocytes that correlate with IAV-induced disease severity [10, 14, 34]. Lung neutrophils release proteases, extracellular traps and reactive oxygen species [40] and create a cytotoxic environment which limits IAV spread at the cost of bystander host cell injury. The same compromise exists for lung monocytes, which directly contribute to IAV-induced morbidity and mortality yet are also required for the priming of effective anti-IAV CD8^+^ T cell responses [14, 38]. In our study, SSa treatment partially attenuated lung neutrophil and monocyte recruitment in response to lethal PR8 infection without impairing systemic neutrophil and monocyte development. Attenuated lung neutrophil responses were also observed following sublethal X-31 infection whilst AMΦ numbers were unaltered following either PR8 or X-31 infection, in agreement with their conversely protective effects following IAV infection [41]. Altogether, our observations suggest that the immunomodulatory effects of SSa treatment is linked to the selective suppression of excessive early lung neutrophil and monocyte numbers, highlighting their potential as a therapeutic target against IAV-induced severe pneumonia.

Many viruses (HIV-1, HBV, HBC, CMV and IAV) can directly activate and appropriate host cell NF-κB signaling to enhance viral replication [16], a pathway similarly activated by oncogenic viruses such as Epstein-Barr virus (EBV) for neoplastic cell transformation [42]. Upon infection, IAV activates a complex network of endosomal and cytosolic pattern recognition receptors (PRRs) such as TLR-3 and TLR-7 (toll-like receptors), and RIG-I and MDA-5 (RIG-I-like receptors) which then activates NF-κB signaling for the induction of pro-inflammatory host cell defence mechanisms [10, 43]. Interestingly, pro-inflammatory NF-κB signaling is preferentially upregulated following IAV sensing compared to anti-viral interferon regulatory factor (IRF)-3 and IRF-7 signaling pathways which are also downstream of TLR and RLR activation [19]. This may reflect a self-beneficial dependency on NF-κB signaling by IAV. Indeed, apart from directly enhancing cRNA promoter dependent vRNA transcription [19], NF-κB activation is also appropriated by IAV to upregulate host cell SOCS-3 expression which suppresses the production of anti-viral IFN-α/β [44]. As heightened NF-κB signaling is also a lethal signature of highly pathogenic IAV strains such as H5N1 [20], the modulation of NF-κB signaling pathways to suppress IAV propagation is an attractive therapeutic target against IAV-induced morbidity and mortality.

ASA (an IκBα kinase inhibitor), BAY11-7085 (an inhibitor of TNF-α induced IκBα phosphorylation) and SC75741 (an inhibitor of NF-κB p65 DNA binding) have all been demonstrated to inhibit IAV replication *in vitro* [17, 18]. Treatment with ASA [18] and SC75741 [45] also inhibited IAV replication in mice although aerosolic ASA administration did not alter IAV-induced mortality or morbidity following lethal PR8 IAV infection similar to our observations. SSa is a lipophilic triterpene saponin derived from *Radix Bupleurum* with established anti-inflammatory and immunomodulatory properties [24–27, 46]. Therapeutically, SSa has been reported to attenuate M-CSF and RANKL-mediated osteoclast generation in bone marrow monocytes chiefly through the inhibition of IκBα phosphorylation (decreasing NF-κB mediated gene transcription) and p38, ERK and JNK phosphorylation (decreasing activation of the mitogen-activation protein kinase pathway; MAP kinase pathway) [24]. The suppression of both pathways by SSa has also been linked to the attenuation of low-grade pro-inflammatory cytokine production in adipocytes [27] and LPS-stimulated RAW264.7 (mouse macrophage) cells [25, 26], in agreement with our own observations that SSa-mediated anti-viral activity occurred through the inhibition of IκBα phosphorylation in IAV-infected A549 cells. As MAP kinase activation is also appropriated by IAV to inhibit protein kinase R dependent suppression of vRNA translation [47], it will be interesting to investigate whether this signaling pathway is also modulated by SSa to confer its anti-viral effects.

*In vitro* and *in vivo* evidence of SSa mediated anti-viral activity was directly demonstrated by virus growth inhibition studies in A549 cells and mouse lung tissue viral titres as well as immunofluorescence studies of caspase-3 associated NP translocation in both settings. Pharmacological inhibition of NF-κB signaling is known to decrease IAV replication through both the suppression of caspase-mediated vRNP nuclear export [17–19, 31] as well as vRNA synthesis itself. The former mechanism was suggested by our studies as *in vitro* SSa treatment decreased nuclear translocation of IAV NP and inhibited IAV-induced cleavage of PARP, a classical substrate for caspase 3, following H5N1 infection in A549 cells. Critically, attenuated cleaved caspase 3 expression was also observed in BAL cells from SSa but not PBS control treated B6 mice, and this was linked to a trend in attenuated total IAV NP expression. Currently, the mechanistic link between caspase 3 activation and vRNP nuclear export remains unclear, although caspases have been demonstrated to disrupt the stability of nuclear pores restricting protein nuclear entry [48, 49], which would in turn allow the nuclear export of viral RNPs.

Critically in this study, SSa treatment protected against lethal PR8 IAV-induced disease morbidity and mortality. PR8 (H1N1) is a highly pathogenic IAV strain which mimics pandemic IAV infections particularly well as both infections can trigger severe primary viral pneumonia (involving extensive airway inflammation, hyaline membrane formation, haemorrhaging, oedema and intra-alveolar and interstitial immune cell infiltration). This severe response is attributed to a combination of rapid IAV replication and enhanced recruitment of pro-inflammatory lung immune cells, notably neutrophils and monocytes, which release pro-inflammatory mediators such as TNF-α and IL-6 [11, 13]. Lung neutrophil and monocyte recruitment and pro-inflammatory lung cytokine production were both attenuated in SSa but not PBS control treated B6 mice following PR8. SSa treatment suppressed IFN-γ, IL-6 and early TNF-α but not IL-1β or IL-10 production in PR8 infected mice, which is advantageous as the latter are linked to the generation of protective adaptive immune responses against IAV [50] and the resolution of inflammation [51]. Lung monocytes are prominent sources of pro-inflammatory cytokines TNF-α and IL-6 [37, 52], which may explain why pro-inflammatory cytokines such as IL-6 are positively correlated, but not causative, to IAV lethality [34]. However, as viral titres are also positively correlated with pro-inflammatory cytokine levels, a secondary reduction in pro-inflammatory cytokine produced subsequent to enhanced anti-viral activity cannot be ruled out in our study. Interestingly, though also disappointingly, administration of SSa more than 72 hours post IAV infection abolished the protective effects of SSa against PR8 infection, suggestive of early anti-viral and/or pro-inflammatory events (< day 3-4 post-infection) as the main targets of SSa treatment.

The extraction of other bioactive compounds from plant-derived indigenous remedies is an area of potential pharmacological gain. Other bioactive constituents of *Radix Bupleurum* include Saikosaponins B, C and D, the mitogenic pectic polysaccharide Bupleuran 2IIc [22] and several lignans, flavonoids and essential oils [23]. Saikosponin B2 has been demonstrated to directly inhibit hepatitis C virus entry and cell membrane fusion [53] whilst Saikosponin D is also associated with the inhibition of NF-κB dependent pro-inflammatory cytokine production [26, 54] and T cell activation [55]. Some conflicting reports exist, however, over whether Saikosponin A and D are bona fide broad-spectrum inhibitors of inflammation-associated immune cells or can conversely also activate residential tissue macrophage and dendritic cell populations [56, 57]. In our studies, SSa administration attenuated lung neutrophil and monocyte recruitment without altering residential AMΦ numbers, suggesting a more selective inhibition of pro-inflammatory innate immune cell recruitment. However, more thorough dissections of the therapeutic effects of Saikosponin A and D on residential immunomodulatory immune cell populations (such as tissue macrophages and regulatory T cells) compared to pro-inflammatory innate immune cells will be beneficial, especially as the former cell types are critical for immune cell and organ homeostasis. This is important as constitutive NF-κB signaling also activates aberrant pro-inflammatory and anti-apoptotic pathways promoting chronic inflammation and tumorigenesis [58], especially in relation to oncogenic viruses, and novel inhibitors of NF-κB may be therapeutically beneficial for diverse indications at doses which minimise disruptions in homeostatic immune cell signaling and other adverse effects.

In summary, we have demonstrated that SSa effectively attenuates IAV replication, including that of a highly pathogenic H5N1 IAV strain *in vitro*, through the inhibition of NF-κB signaling and caspase 3 dependent NP nuclear translocation. SSa administration also protected against lethal PR8-induced mortality and morbidity *in vivo* through the attenuation but not complete abrogation of lung neutrophil and monocyte recruitment as well as decreases in IAV replication, lung tissue pro-inflammatory cytokine production and caspase 3 associated NP nuclear translocation. To our knowledge, this is the first comprehensive report of the anti-IAV efficacy of SSa and our study demonstrates that SSa may possess therapeutic potential especially in the treatment of high pathogenicity influenza virus infections.

## MATERIALS AND METHODS

### Cell lines and IAV strains

A549 cells (a human alveolar adenocarcinoma cell line), Madin-Darby canine kidney cells (MDCK) and MCF-7 cells (a human breast adenocarcinoma cell line) were cultured in Dulbecco's Modified Eagle's Medium (DMEM, Gibco, USA) containing 10% fetal calf serum (FCS) with 100 U/ml of penicillin and 100 μg/ml of streptomycin at 37°C with 5% CO_2_.

Influenza A/Puerto Rico/8/34 (PR8, mouse passaged H1N1) virus was obtained from the Chinese Centre for Disease Control and Prevention (Beijing, China) and from (Melbourne, Australia), respectively. Avian influenza A/Duck/Guangdong/99 (H5N1) virus and A/Chicken/Guangdong/v/2008 (H9N2) virus were kindly provided by the Veterinary Technology Centre of South China Agricultural University (Guangzhou, China). Influenza A/X-31 (X-31, mouse passaged H3N2) virus was kindly provided by Prof Lorene Brown (Melbourne, Australia). New viral stocks of IAV were passaged in 10 day old embryonic chicken eggs for 48 to 72 h. Allantoic fluid was collected and stored at −80°C until required. Experiments involving H5N1 virus strains were conducted in a physical containment level three (PC3) laboratory.

### Mice

Female C57BL/6 (B6) mice were purchased from Guangdong Medical Laboratory Animal Center (Nanhai, Guangdong, China) and the Walter Eliza Hall Institute of Medical Research (Kew, Melbourne, VIC, Australia) respectively. Mice were housed in specific pathogen-free (SPF) isolators. Experiments were performed in mice 8-12 weeks old and respectively approved by the Institutional Animal Care and Use Committee at South China Agricultural University and the La Trobe University Animal Ethics Committee in accordance with the National Health and Medical Research Council Australia code of practice for the care and use of animals for scientific purposes.

### Materials

SSa (purity ≥ 98%) was obtained from Chengdu Pufei De Biotech Co., Ltd (Sichuan, China). Acetylsalicylic acid (ASA) was purchased from Sigma Chemical Co. (St. Louis, MO, USA). Trypsin, TPCK from bovine pancreas and 3-[4,5-dime-thylthylt-hiazol-2-yl]-2,5-diphenyl-tetrazolium bromide (MTT) were obtained from Sigma Chemical Co. (St. Louis, MO, USA). DMEM and FCS purchased from Hyclone Laboratories, Thermo Scientific Co. (Utah, USA). For *in vitro* experiments, SSa and ASA were dissolved in dimethylsulfoxide (DMSO) and diluted with DMEM to < 0.4% DMSO. For *in vivo* experiments, SSa was prepared as oil emulsions consisting of 50 mg SSa dissolved in 200 μl DMSO, followed by the addition of 4.75 ml olive oil and 50 μl Tween 80. 5 ml of sterile endotoxin free water was then gradually added to the solution in an ultrasonic bath to a final SSa stock concentration of 5 mg/ml. PBS control solutions were similarly prepared as oil emulsions using starting volumes of 50 μl PBS mixed in 200 μl DMSO. ASA oil emulsions were also similarly prepared as SSa oil emulsions using starting amounts of 50 mg aspirin dissolved in 200 μl DMSO.

### Virus growth inhibition assay

The virus growth inhibition assay was performed to compare the *in vitro* inhibition of IAV replication by all drug compounds. A549 cell monolayers were infected with 100 TCID_50_ of influenza virus (to ensure a consistent tissue culture infectious dose for each IAV strain) and incubated for 2 h at 37°C. Supernatants were removed and media containing different concentrations of each drug compound then added. Cells and supernatants were then collected at 8, 24, 48, 72 h post-infection and in total subjected to three freeze-thaw cycles at −80°C and 37°C respectively to ensure maximal release of cellular virions. Final supernatant viral titres were determined by the end point dilution assay using MDCK cells and expressed as log_10_ TCID_50_/0.1ml [60]. The IC_50_ value (concentration of compound required to inhibit progeny viral titres by 50%) was determined by plotting the % inhibition of progeny viral titres as a function of compound concentration.

### Immunoblotting

For detection of NF-κB (p65 subunit) levels in the cytosol and nucleus, A549 cells were infected with H5N1 IAV (MOI = 0.1) for 1 h and then cultured in the presence of SSa or DMEM media control. After 24 h incubation, A549 cells were harvested and cytosolic and nuclear proteins separated using a Nuclear-Cytosol Extraction Kit (Beyotime Institute of Biotechnology, China). For detection of total cell IκBα and poly-(ADP-ribose)-polymerase (PARP) levels, A549 cells were lysed in Triton X-100 sampling buffer and protein yields measured using a protein dye (Bio-Rad Laboratories, USA). Equal amounts of protein were separated by SDS-polyacrylamide gel electrophoresis and blotted on nitrocellulose membranes (Millipore, USA). Proteins were detected using mouse or rabbit monoclonal antibodies. β-Actin rabbit mAb (D6A8), NF-κB p65 rabbit mAb (D14E12) [61] and IκBα mouse mAb (112B2) [62] were obtained from Cell Signaling Technology (USA), and PARP-1 mouse mAb (5A5) [63] and Lamin B1 mouse mAb (8D1) were purchased from Santa Cruz Biotechnology (USA). Protein bands were visualized using enhanced chemiluminescence (Thermo Scientific, USA).

### Cell immunofluorescence studies

A549 cells grown on 15 mm cover slips (MatTek, USA) were infected with H5N1 virus at 0.1 MOI for 1 h and treated with SSa or media control. At indicated time points, cells were fixed with 4% paraformaldehyde (PFA) in PBS for 10 min, permeabilized with 0.25% Triton-X100 for 15 min, then either stained with a mouse monoclonal antibody against the influenza A virus nucleoprotein (NP) (clone #65, Immune Technology Corp, USA) or a rabbit monoclonal antibody against NF-κB p65 (D14E12) (Cell Signaling Technology Co., USA) in 1:200 dilution at 4°C for 1 h. Cells were then stained with Alexa Fluor 488 conjugated goat anti-mouse IgG (Cell Signaling Technology Co., USA, 1:1000 dilutions). Cell nuclei were stained using 4′, 6-diamidino-2-phenylindole (DAPI: 1 μg/ml) (Sigma Chemical Co., USA). Cells were analysed using a confocal laser scanning microscope (Zeiss, LSM 710, Germany).

Mouse BAL cells were collected as previously described [37] and 100,000 cells per chamber (in 100 μl RPMI + 10% FCS) were incubated for 30 mins in a 8 well μ-Slide chamber (ibidi) at 37°C, 5% CO2 before fixation (4% PFA, 10 mins RT, 3 × 5 min PBS rinses). Cells were then incubated with surface immune cell 1° Abs where relevant (eBioscience rat anti-mouse CD11b PeCy7 at 1:600 and BD Biosciences hamster anti-mouse CD11c biotin at 1:300; 1 h RT) and then goat anti-rat AF555 (Life technologies, 1:600) and streptavidin AF488 (Life technologies, 1:300) respectively for 30 mins RT. For intracellular cleaved caspase 3 [64] and NP staining, BAL cells were permeablized (0.2% saponin, 20 mins RT), blocked with 10% goat serum (Jackson laboratories, USA, in 0.2% saponin + 1% BSA, 1 h RT), then incubated with 1° Abs (purified rabbit anti-mouse cleaved caspase 3 at 1:300, Cell Signaling; anti-NP mouse ascites at 1:1400, 2 h RT followed by 4 × 5 min in 0.2% saponin + 1% BSA washes) and then 2° Abs (goat anti-rabbit AF647 at 1:600, Life technologies and goat anti-mouse AF488 at 1:600, Life technologies; 1 h RT). Cells were then washed (4 × 5 min in 0.2% saponin + 1% BSA washes) and incubated in 2 μg/ml Hoerchst 33342 (Life technologies, 10 mins RT) before imaged in PBS. All images of were captured on a Zeiss LSM 780 laser scanning confocal microscope (Carl Zeiss AG). Images were visualised and quantified using Imaris (Bitplane AG) and image contrast was increased consistently across all samples was figure visualisation.

### Influenza A virus infection and SSa treatment *in vivo*

For lethal PR8 infections, B6 mice were infected with 500 pfu of PR8 (in 30 μl PBS) intranasally under methoxyflurane anaesthetic. Mice were then subcutaneously injected (27G needle) with SSa, ASA or PBS oil emulsion once a day for 6 consecutive days starting from 4 h post-infection. Mice were daily weighed and monitored for adverse symptoms and culled when weight loss exceeded 25%.

### Lung histopathology

Mouse lungs were inflation fixed in 10% formalin using a hand-held 5 ml syringe, and paraffin embedded. 4-6 μm thick whole lung sections were obtained and stained with H&E. Photomicrographs of stained sections were captured using x20 objectives (Zeiss, AXidskopz).

### Lung tissue viral titres

Mouse lungs were removed, rinsed in PBS, weighed, and homogenized in 1 ml of DMEM with P/S (100 U/ml penicillin and 0.1 mg/ml streptomycin) (Invitrogen, Carlsbad, CA) and centrifuged at 3200g for 5 min. Supernatants were then harvested for viral titre determination using the end point dilution assay in MDCK cells as described above.

### Flow cytometry

Mouse BAL cells were collected as previously described [37], filtered through a 40 μm nylon mesh and resuspended in FACS buffer (Mouse PBS, 2% FCS, 2 mM EDTA). Bone marrow (BM) and splenic cells were harvested through the flushing of femurs and extrusion of splenic cells with RPMI + 10% FCS using a 21G and 23G needle respectively, before single cell resuspension in FACS buffer. Cells were first incubated with Fc block (0.6 μg/ml, anti-mouse CD32/CD16, clone 2.4G2, BD Biosciences) for 10 min on ice. Cells were then stained with primary mAbs or isotype controls (Supplementary Table 1) on ice for 20 min, washed with FACS buffer and resuspended in 120 μl FACS buffer with 5 μl DAPI solution (1 μg/ml, Sigma Aldrich) before analysis. All FACS data were acquired on a BD FACSCanto II (BD Biosciences), and ≥ 50,000 live events collected per sample. Analysis was performed using FlowJo software (Windows V10, FlowJo LLC). Our flow cytometry gating strategy for innate immune cells has been previously described [65].

### Cytokine detection

The level of cytokines IFN-γ, TNF-α, IL-1β, IL-6, IL-10 and MIP-1α in BAL were detected using ELISA Kits (Si-Zheng-Bo, Beijing China) following manufacturers' instructions.

### Statistical analysis

All values are expressed as mean ± SEM from at least three independent experiments. The unpaired two-tailed Student's *t* test was performed for all studies comparing two sets of data. Studies comparing three sets of data were analyzed by the two way ANOVA test followed by multiple comparisons between different treatment groups for statistical significance. The survival rate was analyzed using the χ2 test. Statistical analyses were performed using GraphPad Prism 6.

## SUPPLEMENTARY MATERIAL FIGURES AND TABLE



## Abbreviations

Ab: antibody
ASA: acetylsalicylic acid
AMΦ: alveolar macrophage
BFA: brefeldin A
IAV: influenza A virus
DMEM: Dulbecco's modified eagle's medium
DMSO: dimethylsulfoxide
FBS: fetal bovine serum
ICS: intracellular cytokine staining
IL: interleukin
IFN: interferon
KC: keratinocyte-derived chemoattractant
MDCK: Madin-Darby canine kidney cells
MIP: macrophage inflammatory protein
NF-κB: nuclear factor kappa-light-chain-enhancer of activated B cells
NP: nucleoprotein
PR8: Influenza A/Puerto Rico/8/34 (H1N1)
siRNA: small interfering RNA
SSa: Saikosaponin A
TNF: tumour necrosis factor
X-31: Influenza A/X-31 (H3N2)
