# Supplementary material for: Saikosaponin A inhibits influenza A virus replication and lung immunopathology
Source: Oncotarget. 2015 Dec 2;6(40):42541–56. doi: 10.18632/oncotarget.6448 (PMC4767451; doi:10.18632/oncotarget.6448)
Supplement: Supplementary file 1 [file oncotarget-06-42541-s001.pdf]

Saikosaponin a inhibits influenza A virus replication and lung immunopathology

Supplementary Material

Supplementary methods:

Cell viability assessment using the MTT assay

Monolayers of A549 cells were cultured in 96-well plates and incubated with serial concentrations of SSa for 48 h at 37°C in a humidified 5% CO_2 incubator. Supernatants were removed and 20 µl of MTT (5 mg/ml in serum-free DMEM) was added. After 4 h, excess MTT was removed and formazan crystals solubilized in 150 µl of DMSO. The optical density was measured at 490 nm with a microplate reader (ELX800-UV, Biotex Co., USA). Cell viability was expressed as a percentage of vehicle control and the 50% cytotoxic concentration (CC_{50}) of SSa for A549 cells calculated as the drug concentration required to decrease cell viability by 50%.

***In vivo* SSa toxicity**

To determine *in vivo* SSa toxicity levels, B6 mice were subcutaneously injected (27G needle) with SSa oil emulsions once a day for 6 consecutive days (with 50, 100 and 200 mg/kg/d of SSa respectively). Body weights were determined prior to the first treatment course and for the next 15 days post-treatment.

SSa administration kinetics

B6 mice were subcutaneously injected (27G needle) with SSa oil emulsions (25 mg/kg/d) once a day for 6 consecutive days either beginning at 24 h prior to or 4, 24 or 72 h post-IAV infection. Mice were monitored daily for the next 21 days post-infection.

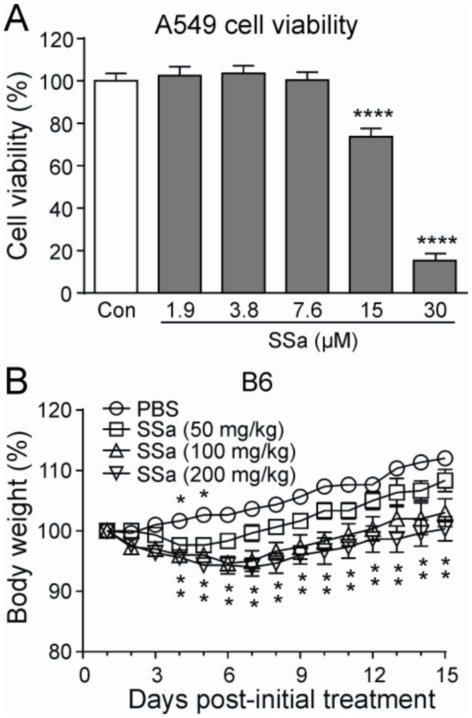

Supplementary Figure 1: ***In vitro*** **and** ***in vivo*** **profile of SSa cytotoxicity**

(A) Cell viability of A549 cells 48 h post-incubation with SSa determined using the MTT assay and expressed as a percentage of the vehicle control (Con, 0.4% DMSO in DMEM). Data is expressed as mean ± SEM of three independent experiments. $^{****}P < 0.0001$ compared to vehicle control. **(B)** Mouse body weights following daily subcutaneous administration of SSa for 6 consecutive days (n = 3). * denotes $P < 0.05$ between 50 mg/kg/d (top), 100 mg/kg/d (middle) or 200 mg/kg (bottom) SSa administered B6 mice compared to PBS controls.

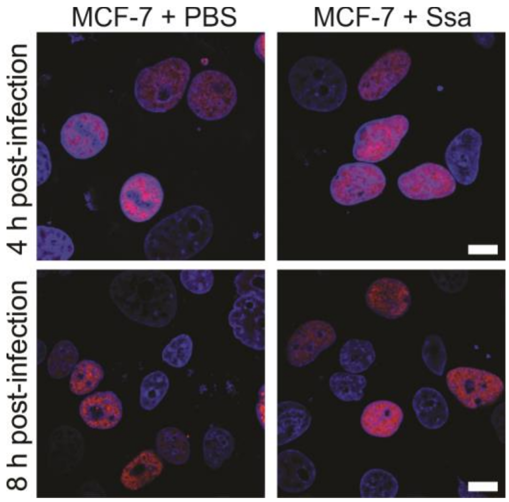

Supplementary Figure 2: vRNP export is dependent on Caspase 3
MCF-7 cells which do not express Caspase 3 were infected with PR8 (MOI = 1, 1 h) and cultured ± 7.6 μM SSA for 4 and 8 h post-infection, fixed and stained for IAV NP expression (NP: red, DAPI nuclear stain: blue, merge: magenta, scale = 10 μm).

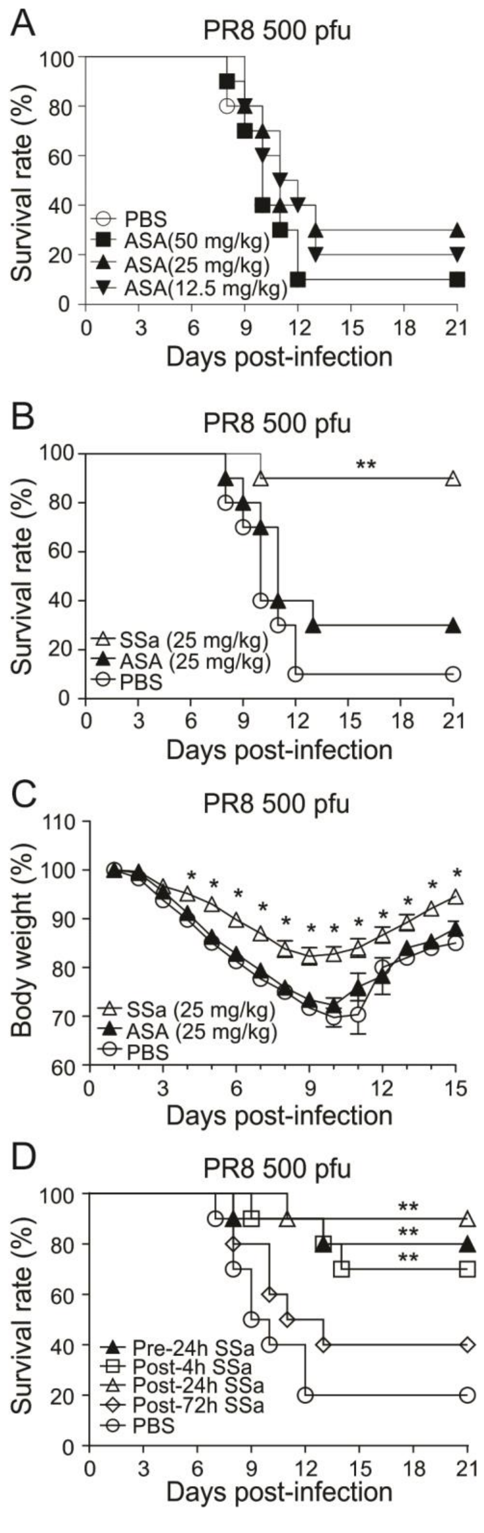

Supplementary Figure 3: Effects of ASA and SSa on PR8 mortality and morbidity
(A) B6 mice were infected with 500 pfu of PR8 and administered ASA or PBS oil emulsions daily (25 mg/kg/d) for 6 days beginning at 4 h post-infection (n = 5-6). **(B)** Effect of SSa versus ASA on survival rates in PR8-infected B6 mice (n = 5-10). PBS oil emulsions used as vehicle controls. $^{**}P<0.01$ with respect to ASA or PBS control. **(C)** Effect of SSa versus ASA on total

body weight loss in PR8-infected B6 mice (n = 5-10). $^{*}P < 0.05$ for SSa with respect to ASA or PBS control. (**D**) Survival rates for PR8-infected B6 mice treated with 25 mg/kg/d SSa for 6 consecutive days beginning at either 24 h prior to or 4, 24 or 72 h post-infection (n = 10). PBS control group was treated at 4 h post-infection. $^{**}P < 0.01$ compared to PBS control.

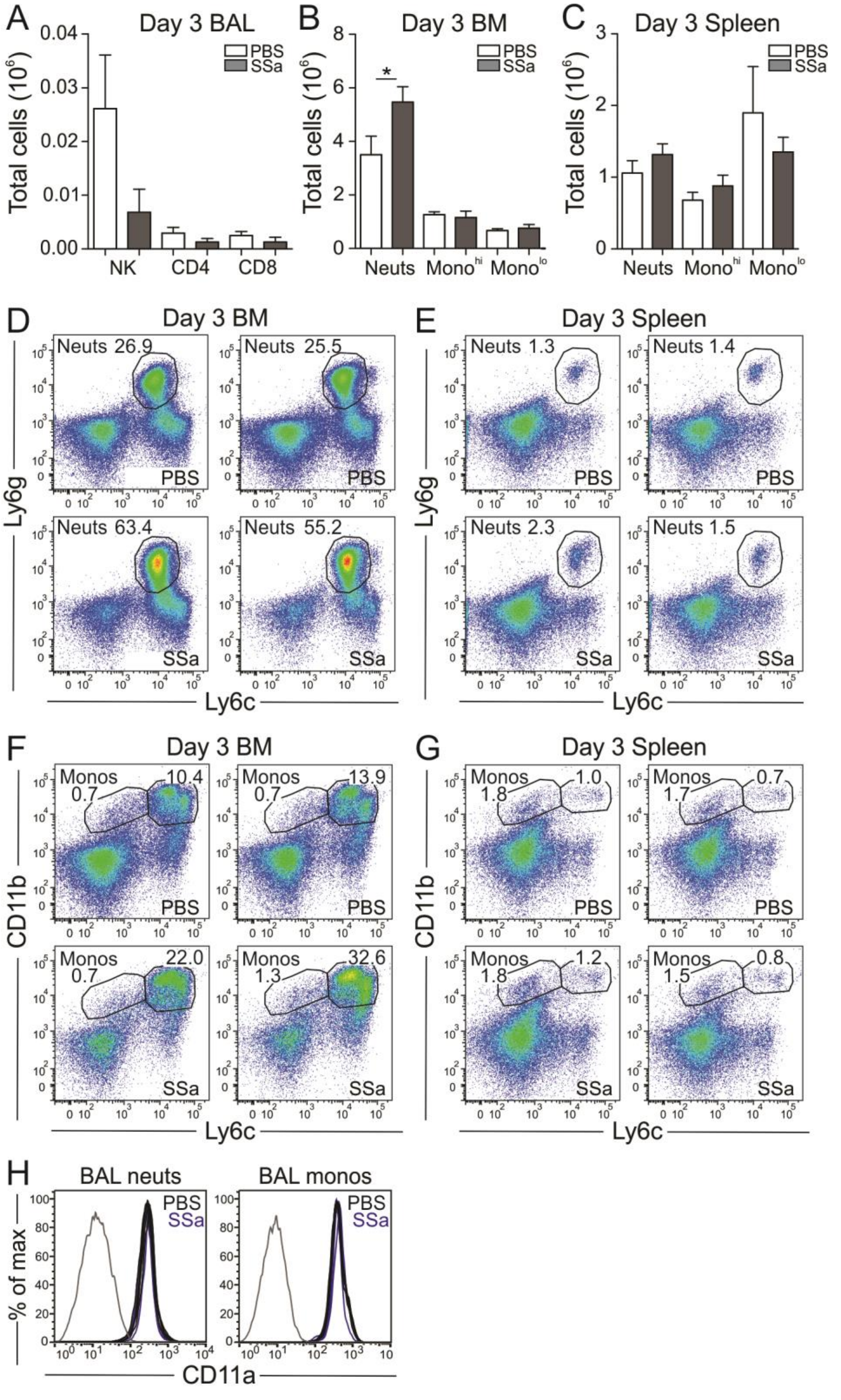

Supplementary Figure 4: Systematic effects of SSa on innate immune cells at day 3 post-PR8 infection

(A) Total NK cell, $CD4^+$ and $CD8^+$ T cell numbers in the BAL of day 3 PR8-infected B6 mice administered SSa or PBS control (25 mg/kg/d), given as daily subcutaneous injections for 6 days

beginning at 4 h post-infection (n = 8). NK cell, $CD4^+$ and $CD8^+$ T cell proportions calculated using flow cytometry. **(B)** Total bone marrow (BM) and **(C)** splenic neutrophil and monocyte ($Ly6c^{high}$ versus $Ly6c^{low}$ subsets) numbers in day 3 PR8-infected B6 mice administered SSa or PBS control (25 mg/kg/d, n = 8). * $P < 0.05$ for SSa treated mice with respect to PBS control. **(D)** BM and **(E)** splenic neutrophil proportions of all $CD45^{pos}$ leukocytes in day 3 PR8-infected B6 mice administered SSa or PBS control (25 mg/kg/d). Representative of two independent experiments (n = 8). **(F)** BM and **(G)** splenic $Ly6c^{high}$ versus $Ly6c^{low}$ monocyte proportions of all $CD45^{pos}$ leukocytes in day 3 PR8-infected B6 mice administered SSa or PBS control (25 mg/kg/d). Representative of two independent experiments (n = 8). **(H)** BAL neutrophil and monocyte surface CD11a expression in day 3 PR8-infected B6 mice administered SSa or PBS control (25 mg/kg/d, n = 4; black = PBS, blue = SSa, grey = non-binding Ab control).

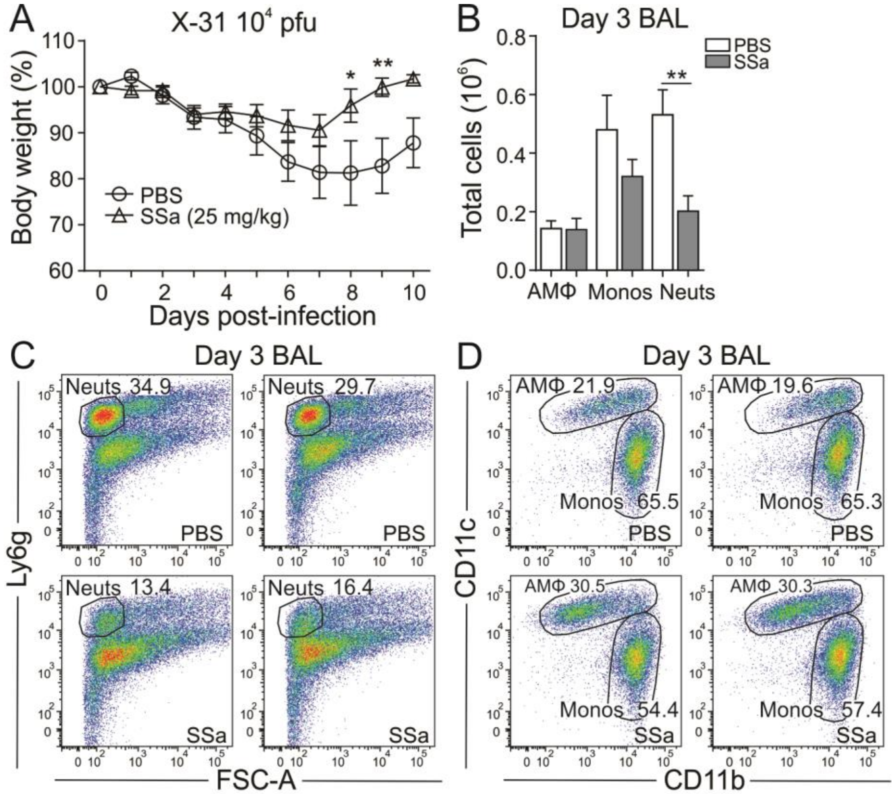

Supplementary Figure 5: SSa attenuates lung neutrophil recruitment in X-31 infected mice
(A) Effect of SSa (25mg/kg/d) on total body weight loss in X-31-infected B6 mice (n = 5). $^{*}P < 0.05$ and $^{**}P < 0.01$ for SSa with respect to PBS control. SSa administered as daily subcutaneous injections for 6 days beginning at 4 h post-infection. **(B)** AMΦ, monocyte and neutrophil numbers in the BAL of day 3 X-31-infected B6 mice administered SSa or PBS control (25 mg/kg). AMΦ, monocyte and neutrophil BAL cell proportions calculated using flow cytometry. $^{*}P < 0.05$ for SSa with respect to PBS control. **(C)** BAL neutrophil and **(D)** AMΦ and monocyte proportions in day 3 X-31-infected B6 mice administered SSa or PBS control (25 mg/kg/d). Representative of n = 5; two independent experiments.

Supplementary Table 1: Monoclonal antibodies used for mouse flow cytometry studies

Antibody	Fluorophore	Isotype	Dilution	Company
CD45	FITC	Rat IgG1, κ	1:300	BD Biosciences
Ly6g	PE	Rat IgG2b, κ	1:300	BD Biosciences
CD11c	APC	Ar Ham IgG1, λ2	1:300	BD Biosciences
CD11b	PE-Cy7	Rat IgG2b, κ	1:600	eBioscience
Ly6c	APC-eFluor 780	Rat IgG2c, κ	1:300	eBioscience
CD11a	PerCP-Cy5.5	Rat IgG2a, κ	1:300	Biolegend
CD115	PE	Rat IgG2a, κ	1:300	eBioscience
CD3	APC	Ar Ham IgG	1:300	eBioscience
CD4	Pe-Cy7	Rat IgG2a, κ	1:300	BD Biosciences
CD8a	BV510	Rat IgG2a, κ	1:300	BD Biosciences
Isotype control	PE	Rat IgG1, κ	1:300	BD Biosciences
Isotype control	APC	Rat IgG2b, κ	1:300	BD Biosciences
Isotype control	PerCP-Cy5.5	Rat IgG2b, κ	1:300	Biolegend
